# *Auricularia auricula’s* Polysaccharides Confer Protection in Murine Sepsis via Immune Modulation

**DOI:** 10.1155/jimr/9938963

**Published:** 2026-08-25

**Authors:** Jesse Pereira Machado Viana, Luisa Coutinho Coelho, Priscila M. Mendes, André Alvares Marques Vale, Jeferson Noslen Casarin, Joicy Cortez de Sá Sousa, Paulo Vitor Soeiro Pereira, Maria Carolina B. Di Medeiros Leal, Anamelia L. Bocca, Márcia C. G. Maciel

**Affiliations:** ^1^ Cell Biology Department, University of Brasília, Brasília, Federal District, Brazil, unb.br; ^2^ Graduate Program in Health Sciences, Federal University of Maranhão, São Luis, Maranhão, Brazil, ufma.br; ^3^ IDEA School of Medicine, São Luís, Maranhão, Brazil; ^4^ Graduate Program in Health and Technology, Federal University of Maranhão, Imperatriz, Maranhão, Brazil, ufma.br; ^5^ Postgraduate Program in Pharmaceutical Sciences, Pharmacology and Therapeutics (PPGCFFT), Universidade Evangélica de Goiás (UniEVANGÉLICA), Anápolis, Goiás, Brazil; ^6^ Center for Molecular Biotechnology (C-Biotech), University of Brasília Science and Technology Park (PCTec), Asa Norte, Brasília 70910-900, Distrito Federal, Brazil; ^7^ Department Bi-Institutional Translational Medicine Platform, Oswaldo Cruz Foundation (Fiocruz), Ribeirão Preto 14049900, Brazil, fiocruz.br; ^8^ Center of Molecular Biotechnology (C-BIOTECH), University of Brasília, Brasília, Federal District, Brazil, unb.br

**Keywords:** beta-glucan, cytokines, infection, inflammation, polysaccharides, prebiotic, survival

## Abstract

Sepsis is a life‐threatening condition characterized by severe organ dysfunction resulting from an uncontrolled host response to infection. Sepsis treatment poses a challenge due to its complexity and the need for effective therapeutic approaches. The objective of this study was to evaluate the immunomodulatory and antimicrobial effects of exopolysaccharides (EPS) from *Auricularia auricula* in a murine model of lethal sepsis. Our findings demonstrated that treatment with EPS significantly enhances the proliferation of circulating lymphocytes and granulocytes, which is associated with improved host defense mechanisms. Additionally, EPS administration effectively mitigates sepsis‐induced pulmonary damage, as evidenced by preserved lung function. Furthermore, EPS treatment significantly reduces microbial translocation to the bloodstream, maintaining a lower colony‐forming unit (CFU) count and thereby restricting systemic infection. The polysaccharides also prevent the development of thrombocytopenia in septic animals, thereby preserving platelet homeostasis. The compound modulates serum cytokine profiles, contributing to a more regulated inflammatory response. The interactome analysis reveals that the Dectin‐1 → Syk pathway activates NF‐κB/AP‐1 and involves p38 MAPK, thereby explaining the observed cytokine modulation. The presence of Nod2/Ripk2 suggests that the EPS primes leukocytes to effectively combat both fungi and bacteria, leading to a reduced bacterial load and increased survival. Collectively, these data indicate that EPS, a prebiotic agent, represents a novel therapeutic strategy with potential for modulating immune responses, controlling microbial proliferation, and improving survival outcomes in a model of severe sepsis.

## 1. Introduction

Sepsis is a potentially fatal organ dysfunction resulting from a dysregulated host response to infection [1]. This definition was the complex and heterogeneous nature of the phenomenon, highlighting that an uncontrolled host response can eventually lead to multiple organ dysfunction syndrome due to various complications. Furthermore, an exacerbated inflammatory response can precipitate septic shock. This critical condition arises when a patient becomes unresponsive to fluid resuscitation, developing persistent hypotension accompanied by cellular and metabolic dysfunctions. These dysfunctions significantly increase the risk of mortality in sepsis [2].

Sepsis poses a severe public health problem due to its high incidence, mortality rates, and significant treatment costs [3]. The mortality rate from sepsis is particularly high in low‐ and middle‐income countries, where limited resources and inadequate medical infrastructure exacerbate the issue. Moreover, it’s estimated that one in three cases of sepsis annually results in death [4].

A variety of pathogens, including bacteria, viruses, fungi, and protozoa, can cause sepsis in humans, with bacteria being the predominant culprits [1]. Among the primary bacterial agents, *Staphylococcus aureus*, *Escherichia coli*, and *Streptococcus pneumoniae* are particularly notable, in addition to multidrug‐resistant pathogens [5, 6]. The increasing antimicrobial resistance further complicates the treatment of sepsis. When the causative pathogens are resistant to multiple medications, therapeutic options become limited, leading to less effective treatments, more prolonged and severe infections, and ultimately, higher mortality rates. Moreover, the direct and indirect costs associated with sepsis treatment impose a significant burden on healthcare systems. This includes extended intensive care, often requiring ventilatory support and surgical interventions, leading to prolonged stays in intensive care units [7]. Beyond acute care, substantial costs are also associated with rehabilitation and long‐term care as patients frequently suffer from lasting physical, cognitive, and emotional sequelae [8, 9]. Thus, the combination of high mortality, growing antimicrobial resistance, and the substantial economic burden positions sepsis as a critical global health challenge [10]. This scenario underscores the urgent need for novel alternative and/or complementary therapies that can mitigate the infectious agent’s load on the host as well as strategies to modulate the immune response, ultimately aiming for a better prognosis.

In this context, polysaccharides emerge as a promising class of compounds for treating infections given their natural and easily obtainable nature. Found in the cell walls of fungi, yeasts, cereals, and algae, polysaccharides are well‐known for their immunomodulatory properties [11], exhibiting prebiotic activity that promotes the growth of beneficial bacteria in the gut microbiota while inhibiting the proliferation of undesirable pathogens. Thus, polysaccharide components play a crucial role in maintaining the balance of the gastrointestinal microbiota [12]. A systematic review of randomized clinical trials investigates the effect of exclusive oral administration of fungal beta‐(1 → 3, 1 → 6)‐D‐glucans, in various forms and dosages, on maintaining homeostasis in healthy or susceptible individuals. The review showed that the majority of studies highlighted beneficial effects on immunomodulation and immune system potentiation. A reduction in the incidence and symptoms of common colds, influenza, and upper respiratory tract infections is evidence of these effects [13]. Further supporting these findings. Walachowski et al. [14] evaluated the effects of oral supplementation with purified β‐glucans from *Saccharomyces cerevisiae* in mice infected intraperitoneally with *E. coli*. The results showed improved infection control, as evidenced by a lower bacterial load and reduced tissue damage, including necrosis in the liver and spleen. Another notable effect of yeast β‐glucan treatment is its ability to stimulate monocyte/macrophage activation, promoting increased cytokine secretion during a secondary challenge—a phenomenon termed “immune training” [15]. The authors demonstrated in vitro that monocytes incubated with β‐glucan induced trained immune cells, characterized by increased TNF‐α and IL‐6 secretion. Following an infectious challenge, monocytes can retain the “trained immunity” acquired from a previous encounter, entering a state of either tolerance or heightened responsiveness, especially in response to *Candida albicans* [16]. The exopolysaccharides (EPS) from *Auricularia auricula*, composed of homopolymers characteristic of α and β glucans and heteropolymers consistent with fucogalactomannans, demonstrated score mitigation in the murine colitis model by modulating cytokine secretion and microbiota composition, events dependent on the Dectin‐1 receptor, proving the role of the β‐glucan fraction in the EPS activity [17].

Given the increasing relevance of polysaccharides as therapeutic agents for modulating the immune response, this study aims to investigate the effect of a mushroom‐derived polysaccharide containing a high concentration of β‐glucan in a lethal cecal ligation and puncture (CLP) model of sepsis. Through this investigation, we seek to provide insights into how β‐glucans from *A. auricula* EPS can help mitigate dysregulated inflammatory responses, reduce mortality, and improve clinical outcomes in animals with induced systemic infections. Ultimately, we propose incorporating this polysaccharide into the diets of patients at risk of developing sepsis, suggesting a novel approach to mitigate this life‐threatening condition.

## 2. Materials and Methods

### 2.1. Cultivation of *Auricularia auricula* and Polysaccharide Extraction

The *A. auricula* (CC 309) mushrooms were sourced from the Mushroom Germplasm Bank for Human Use at the Brazilian Agricultural Research Corporation (EMBRAPA), Genetic Resources and Biotechnology Unit—Cenargen, under the coordination of Dr. Arailde Fontes Urben. Access to this genetic heritage was authorized by the National Council for Scientific and Technological Development (CNPq), as documented in the Authorization Term Number 010342/2014‐1.

The basidiomycete *A. auricula* was cultivated on solid medium, Potato Dextrose Agar (Acumedia, Ref. 7585A), prepared according to the manufacturer’s instructions using 10% agar (Kasvi, Ref. K25‐611001) and incubated at 25°C until the mycelium covered the entire plate. A submerged culture method was employed to obtain polysaccharides. An initial inoculum was prepared with mycelium fragments in an Erlenmeyer flask containing 100 mL of potato dextrose medium. The culture was maintained at 30°C with agitation for 7 days. After this period, fresh culture medium was added to the inoculum, and again, it was cultivated under the same conditions. The mycelium was separated by filtration and washed to remove low‐molecular‐weight components. Subsequently, polysaccharides were extracted as described previously [18], then precipitated, centrifuged, and washed with acetone. After washing, the material was resuspended in deionized water and lyophilized before proceeding to further characterization and biological activity assays. The exopolysaccharide (EPS) composition has been previously described [17].

The EPS was not contaminated with lipopolysaccharide (LPS) since the carbon spectrum shows no signal in alpha‐carbonyl carbons at 34 ppm and the carbonyl region at 170–175 ppm, where no characteristic signals of ester or lipid amide carbonyls, nor of the acetyl groups from sugars, are present.

### 2.2. Murine Model of Sepsis

This study utilized female C57BL/6 mice aged 8–12 weeks (average weight: 25 g). The animals were obtained from the bioterium of the Federal University of Uberlândia (UFU) and housed at the Institute of Biological Sciences (ICB) of the University of Brasília (UnB). The mice were housed under controlled conditions with a temperature of 26 ± 2°C, relative humidity of 40%–60%, and a 12 h light/dark cycle and had free access to food and water. The study was conducted in accordance with the guidelines approved by the Animal Use Ethics Committee of the Federal University of Brasília (Protocol: 23115.002346/2005‐45), which considered protocols to minimize pain, suffering, and distress.

They were randomly assigned to one of three experimental groups: the Sham group, which underwent a laparotomy procedure without CLP. CLP group: which was subjected to CLP without any additional treatment. CLP/GLU group: which received prophylactic treatment with EPS (5 mg/kg) via gavage for 15 days before the CLP procedure.

Polymicrobial sepsis was induced using the CLP method described by Benjamim et al. [19], with minor modifications. Briefly, all mice were intraperitoneally anesthetized with a mixture of 100 mg/kg ketamine hydrochloride and 10 mg/kg xylazine hydrochloride. Laparotomy was performed, and the cecum was mobilized, ligated below the cecal valve, and punctured 8 times using an 18‐gauge needle to induce lethal sepsis. The cecum was placed back into the peritoneal cavity, and the abdomen was closed in two layers. Saline (0.5 mL/10 g body weight) was administered subcutaneously to the animals as a hydration fluid.

We conducted two distinct in vivo experiments: a survival analysis and an evaluation of cellular, immunological, and functional parameters. In the survival analysis, the mice were observed every 24 h for a period of 5 days. At the end of the observation period, or if humane endpoints were met, they were euthanized with an anesthetic overdose (150 mg/kg ketamine hydrochloride and 120 mg/kg xylazine hydrochloride) [20]. For the assessment of cellular, immunological, and functional parameters, biological samples were collected 12 h after the CLP procedure. Blood and tissue samples were then analyzed to evaluate their antimicrobial, anti‐inflammatory, and immunological properties [21, 22].

### 2.3. Evaluation of Cellular, Immunological, and Functional Parameters

For the assessment of cellular, immunological, and functional parameters, biological samples were collected 12 h after the CLP procedure. Blood and tissue samples were then analyzed to evaluate their antimicrobial, anti‐inflammatory, and immunological properties [21].

After euthanasia, blood samples were collected from the retro‐orbital plexus into conical plastic tubes precoated with EDTA. A Hematoclin Haematology Analyser 2.8 VET (R666) was used to analyze hematological parameters. These parameters included the percentages of leukocyte subpopulations and their total counts as well as platelet counts.

### 2.4. Determination of Bacterial Burden

To quantify the bacterial load, 10 μL samples of both blood and peritoneal lavage fluid were collected. The blood sample was serially diluted (10^2^ colony‐forming unit [CFU]/mL) by adding 90 μL of phosphate‐buffered saline (PBS) to each well of a 96‐well plate. The peritoneal lavage fluid sample followed the same dilution parameters, with a serial dilution of 10^5^ CFU/mL. Subsequently, 100 μL aliquots of these dilutions were plated onto brain heart infusion (BHI) agar plates (Difco Laboratories, Detroit) and spread using a Drigalski spreader. The CFUs were counted after overnight incubation at 37°C, and results are expressed as CFU/mL [21].

### 2.5. Determination of Cellularity

Following euthanasia, the femur, spleen, and mesenteric lymph nodes were collected. The femur was perfused with 1 mL of ice‐cold PBS to isolate bone marrow cells. The spleen was then mechanically dissociated, washed with 5 mL of PBS, and subsequently passed through a 70 µm cell strainer. Similarly, the mesenteric lymph node was dissociated, washed with 1 mL of PBS, and then passed through a 70 µm cell strainer. Finally, the isolated cells were stained with 0.05% crystal violet (in 30% acetic acid) and counted using a Neubauer chamber under a light microscope at 400× magnification.

Peritoneal cells were collected by lavaging the peritoneal cavity with 5 mL of sterile, ice‐cold PBS. Following collection, the cells were stained with 0.05% crystal violet (in 30% acetic acid) and counted using a Neubauer chamber under a light microscope at 400× magnification.

### 2.6. Cytokine Quantification

Serum cytokine levels were measured using the BD Cytometric Bead Array Mouse Inflammatory Cytokine Kit (Cat. Number 552364). This assay quantified the concentrations of tumor necrosis factor‐alpha (TNF‐α), monocyte chemoattractant protein‐1 (MCP‐1), interleukin (IL)‐6, IL‐10, IL‐12, and interferon (IFN‐γ) in serum samples. The procedure followed the protocol described by Hodge et al. [23]. Samples were analyzed on a FACS Calibur flow cytometer, and data were processed using FCAP Array Version 3.0 software (BD Biosciences, San Jose, CA, USA). Results are expressed in pg/mL for each cytokine.

### 2.7. Histological Analysis

Lungs were removed and fixed in 10% formalin. Tissue samples were then processed through a series of alcohol baths and embedded in paraffin. Thin sections (5 μm) were prepared using a microtome, mounted on slides, and stained with H&E. These sections were examined for circulatory changes, inflammatory infiltrates, necrosis, and edema. Histological images of the lungs were captured using an Aperio scanner and analyzed with Aperio ImageScope Pathology Slide Visualization software. Images were acquired at various magnifications: 1× (2 mm), 2× (1 mm), 4× (500 µm), 5× (400 µm), 10× (200 µm), and 20× (100 µm). The results are expressed as the means ± SD from the scores: 0‐absent, 1‐weak, 2‐moderate, and 3‐strong.

### 2.8. Protein–Protein Interaction Network Analysis

To map the functional interaction networks among proteins within the Dectin‐1 signaling pathway, we utilized STRING v12. This bioinformatics resource integrates data from diverse sources, including public databases and high‐throughput datasets, enabling the creation of detailed maps of the protein interactome. Evidence for protein associations within the STRING database originates from independent sources, referred to as “channels.” These include genomic context information (neighborhood, gene fusion, and gene co‐occurrence), co‐expression, text mining, biochemical/genetic data (“experiments”), and previously curated pathway and protein complex datasets (“databases”) [24]. For our analysis, we queried the default settings for functional interaction networks specific to Dectin‐1 signaling proteins in *Mus musculus*. The association sources employed for identifying functional interactions were text mining, experiments, databases, co‐expression, neighborhood, and co‐occurrence. In the generated network, nodes represent proteins and edges denote protein–protein associations. Interactions were mapped with a medium confidence score (0.400).

### 2.9. Statistical Analysis

Results are expressed as the mean ± standard deviation (SD). The normality of the data was assessed using the D’Agostino–Pearson test. Statistical analysis was performed using ANOVA with Tukey’s post‐hoc test in GraphPad Prism version 8.0.2. Differences were considered statistically significant when *p*  < 0.05. For survival analysis, Kaplan–Meier curves were used, and the log‐rank test was employed to compare them.

## 3. Results

### 3.1. ^13^C Nuclear Magnetic Resonance (NMR) Spectrum Demonstrating the Absence of LPS in the Sample

**Figure 1:**
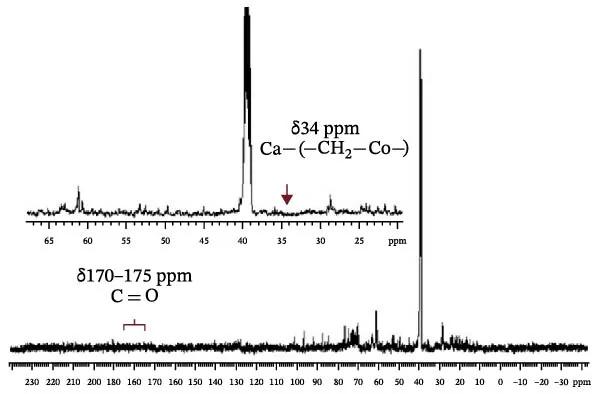
^13^C nuclear magnetic resonance (NMR) spectrum showing the absence of lipopolysaccharide (LPS) in the analyzed sample. Two diagnostic regions were evaluated: (i) at *σ* 34 ppm, corresponding to α‐carbonyl carbons (Ca─CH_2_─CO─), where no detectable signal is observed; and (ii) in the carbonyl region between *σ* 170–175 ppm, where no signals attributable to ester or lipid amide carbonyls or sugar acetyl groups are observed. The absence of these signals, characteristic of the chemical structure of LPS, confirms the absence of this contaminant in the sample.

The EPS was not contaminated with LPS since the carbon spectrum shows no signal in alpha‐carbonyl carbons at 34 ppm and carbonyl region at 170–175 ppm, where no characteristic signals of ester or lipid amide carbonyls, nor of the acetyl groups from sugars, are present (Figure 1).

### 3.2. Effect of EPS Treatment on Peripheral Blood Cell Recruitment in Septic Mice

**Figure 2:**
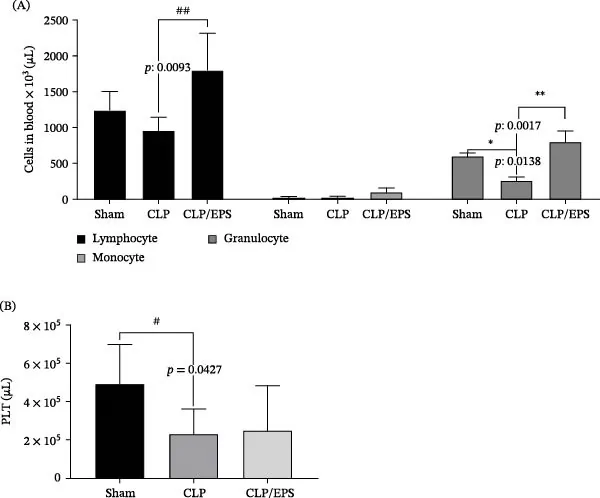
Determination of absolute leukocyte and platelet counts in peripheral blood. The absolute number of leukocytes (A) and platelets (B) in the peripheral blood of the experimental groups. Animals were divided into three groups—Sham: Untreated C57BL/6 mice without sepsis. CLP: Untreated C57BL/6 mice subjected to cecal ligation and puncture (sepsis induction). CLP/EPS: C57BL/6 mice were treated via oral gavage with an EPS solution (5 mg/kg) for 15 days before sepsis induction. Our results show that EPS treatment in septic mice led to a significant increase in circulating lymphocytes (*p* = 0.0093) and granulocytes (*p* = 0.0017) compared to the CLP group. No statistical difference was observed between the Sham and CLP/EPS groups for these cell types. Furthermore, a reduction in platelet count (thrombocytopenia) was observed between the CLP and Sham groups (*p* = 0.0427). Results are expressed as the mean ± standard deviation (SD) (*n* = 5). The normality of the data was assessed using the D’Agostino–Pearson test. Statistical analysis was performed using ANOVA with Tukey’s post‐hoc test in GraphPad Prism Version 8.0.2. Differences were considered statistically significant when *p* < 0.05.  ^∗^: *p* = 0.0138,  ^∗∗^: *p* = 0.0017, ^#^: *p* = 0.0427, ^##^: *p* = 0.0093.

Treatment with EPS in septic mice (CLP/EPS) significantly increased the absolute number of lymphocytes (*p* = 0.0093) and granulocytes (*p* = 0.0017) in peripheral blood when compared to the CLP group (Figure 2A). Conversely, the CLP group exhibited a reduction in circulating granulocytes (*p* = 0.0138) (Figure 2A). Furthermore, the CLP group also presented with thrombocytopenia (*p* = 0.0427) when compared to the Sham group (Figure 2B).

### 3.3. Effect of EPS Treatment on Cellularity of Bone Marrow, Spleen, Lymph Node, and Peritoneum in Septic Mice

The CLP group, when compared with the SHAM group, showed reduced cellularity in the bone marrow (Figure 3A) and an increase in the spleen (Figure 3B) and in the peritoneum (Figure 3C). The EPS treatment significantly reduced the number of cells in the bone marrow compared with the SHAM group, and in other analyses, there was no difference between these groups. When comparing the CLP‐treated and nontreated EPS, there is a decrease in cell numbers in the spleen and peritoneum. No difference in the lymph node cellularity was observed across groups (Figure 3D).

Figure 3Effect of EPS treatment on cellularity of septic mice. Animals were divided into three groups—Sham: Untreated C57BL/6 mice without sepsis. CLP: Untreated C57BL/6 mice with sepsis induced by cecal ligation and puncture. CLP/EPS: C57BL/6 mice treated via oral gavage with a EPS solution (5 mg/kg) for 15 days before sepsis induction. Our results indicate that β‐glucan treatment reduced the number of cells in the bone marrow (A), spleen (B), and peritoneum (C) compared with the CLP group. However, no significant difference was observed in the lymph node (D). Statistical differences were observed between the CLP and Sham groups for bone marrow, spleen, and peritoneum. Results are expressed as the mean ± standard deviation (SD) (*n* = 5). The normality of the data was assessed using the D’Agostino–Pearson test. Statistical analysis was performed using ANOVA with Tukey’s post‐hoc test in GraphPad Prism Version 8.0.2. Differences were considered statistically significant when *p* < 0.05.  ^∗^: *p* = 0.0218,  ^∗∗^: *p* = 0.0078,  ^∗∗∗^: *p* = 0.0002,  ^∗∗∗∗^: *p* < 0.0001, ^##^: *p* = 0.0020.

**Figure:**
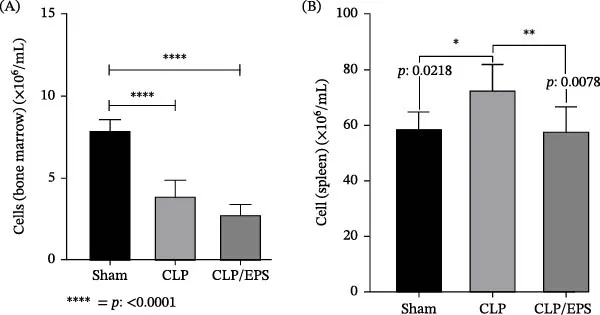

**Figure:**
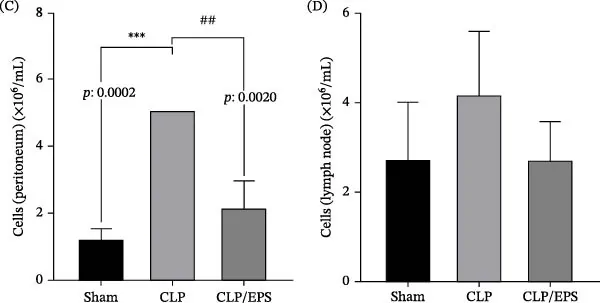

### 3.4. Effect of EPS Treatment on Lung Histology in Septic Mice

Histological analysis was performed to assess congestion, hemorrhage, edema, and inflammation in the lungs of mice from the Sham, CLP, and CLP/EPS groups. Histological analysis revealed distinct profiles across the experimental groups, although the basic tissue architecture remained preserved in all cases.

**Figure 4:**
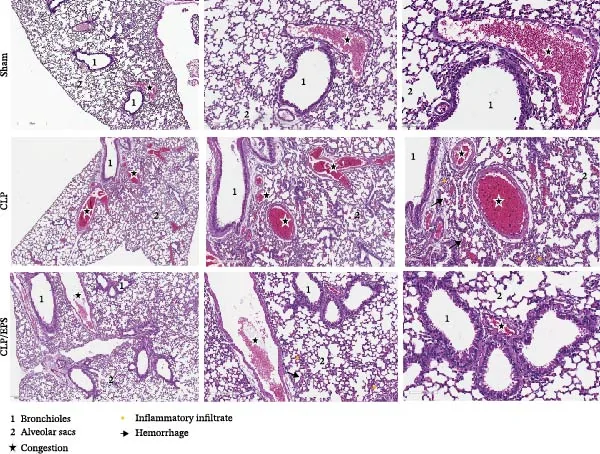
Effect of EPS treatment on lung histology in septic mice. Animals were divided into three groups—Sham: Untreated C57BL/6 mice without sepsis. CLP: Untreated C57BL/6 mice with sepsis induced by cecal ligation and puncture. CLP/EPS: C57BL/6 mice treated via oral gavage with a EPS solution (5 mg/kg) for 15 days before sepsis induction. Images were acquired at various magnifications: 1× (2 mm), 2× (1 mm), 4× (500 µm), 5× (400 µm), 10× (200 µm), and 20× (100 µm). The meaning of the symbols is described in the figure.

In the Sham group, the lung architecture was preserved, with normal bronchioles and alveolar sacs and no signs of edema, inflammatory infiltrates, or interalveolar septal thickening. Congestion occurred in all groups. The untreated CLP group showed intense bleeding, edema, and inflammation when compared to Sham. The CLP/EPS group showed reduced bleeding, edema, and inflammation compared to those of CLP (Table 1 and Figure 4).

**Table 1:** Evaluation of pulmonary inflammation.

	Sham	CLP	CLP/EPS
Congestion	1.8 ± 1.3	2.4 ± 0.8	1.6 ± 0.8
Hemorrhage	0.0 ± 0.0	1.6 ± 0.5^∗∗∗^	0.6 ± 0.5^#^
Edema	0.0 ± 0.0	2.2 ± 0.4^∗∗∗^	0.4 ± 0.8^###^
Inflammation	0.0 ± 0.0	2.2 ± 0.4^∗∗∗∗^	1.0 ± 0.0^####^

*Note:* Animals were divided into three groups: Sham, CLP, and CLP/EPS. *N* = 5 animals per group. The results are expressed as the means ± SD from the scores: 0—absent, 1—weak, 2—moderate, 3—strong. ^#^, when compared to CLP;  ^∗^, when compared to Sham; CLP, untreated C57BL/6 mice with sepsis induced by cecal ligation and puncture; CLP/EPS, C57BL/6 mice treated via oral gavage with a EPS solution (5 mg/kg) for 15 days before sepsis induction; Sham, Untreated C57BL/6 mice without sepsis.

^∗∗∗^ *p* = 0.0003

^∗∗∗∗^ *p* < 0.0001

^#^ *p* = 0.0106

^###^ *p* = 0.0009

^####^ *p* < 0.0001

### 3.5. Effect of EPS Treatment on Serum Cytokine Release in Septic Mice

**Figure 5:**
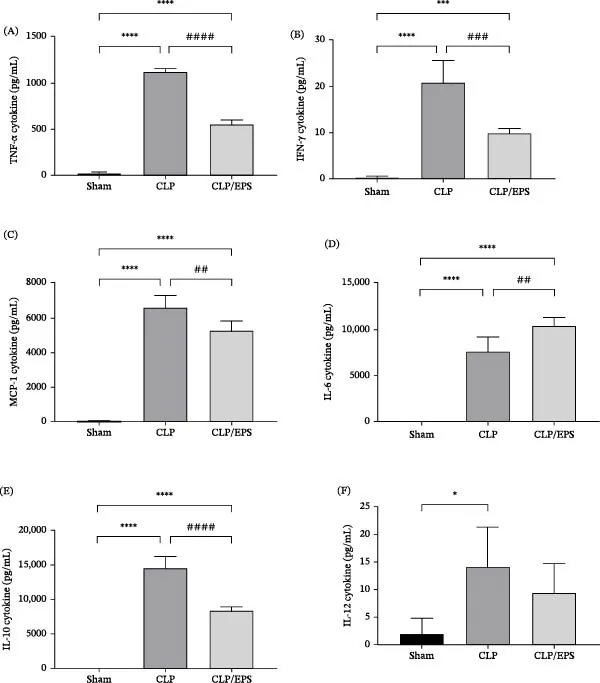
Effect of EPS treatment on serum cytokine release in septic mice. Animals were divided into three groups—Sham: Untreated C57BL/6 mice without sepsis. CLP: Untreated C57BL/6 mice with sepsis induced by cecal ligation and puncture. CLP/EPS: C57BL/6 mice were treated via oral gavage with a EPS solution (5 mg/kg) for 15 days before sepsis induction. After serum collection, levels of TNF‐α (A), IFN‐γ (B), MCP‐1 (C), IL‐6 (D), IL‐10 (E), and IL‐12 (F) were evaluated. Results are expressed as the mean ± standard deviation (SD) (*n* = 5). The normality of the data was assessed using the D’Agostino–Pearson test. Statistical analysis was performed using ANOVA with Tukey’s post‐hoc test in GraphPad Prism Version 8.0.2. Differences were considered statistically significant when *p* < 0.05.  ^∗^: *p* = 0.0100,  ^∗∗∗^: *p* = 0.0005,  ^∗∗∗∗^: *p* < 0.0001, ^##^: *p* = 0.0030, ^###^: *p* = 0.0001, ^####^: *p* < 0.0001.

The CLP‐induced increases in all cytokines compared with the SHAM group (Figure 5). The analysis of serum cytokine levels revealed that EPS treatment significantly reduced systemic TNF‐α (*p*  < 0.0001), MCP‐1 (*p*  < 0.0001), IL‐10 (*p*  < 0.0001), and IFN‐γ (*p* = 0.0005) levels in septic mice compared with the CLP group (Figure 5). However, this treatment also resulted in a significant increase in IL‐6 levels compared with both the CLP group (*p* = 0.0030) and the Sham group (*p*  < 0.0001). Additionally, IL‐12 levels differed significantly only between the CLP and Sham groups (*p* = 0.0100).

### 3.6. Effect of EPS Treatment on Bacterial Burden in Septic Mice

**Figure 6:**
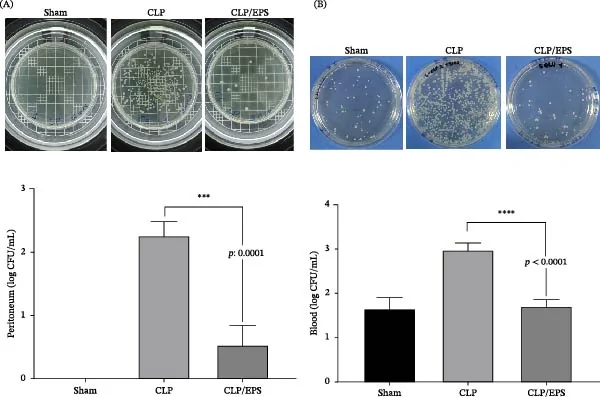
EPS reduced colony‐forming units (CFUs) in the peritoneum (A) and peripheral blood (B). Animals were divided into three groups—Sham: Untreated C57BL/6 mice without sepsis. CLP: Untreated C57BL/6 mice with sepsis induced by cecal ligation and puncture. CLP/EPS: C57BL/6 mice treated via oral gavage with a EPS solution (5 mg/kg) for 15 days before sepsis induction. Results are expressed as the mean ± standard deviation (SD) (*n* = 5). The normality of the data was assessed using the D’Agostino–Pearson test. Statistical analysis was performed using ANOVA with Tukey’s post‐hoc test in GraphPad Prism Version 8.0.2. Differences were considered statistically significant when *p* < 0.05.  ^∗∗∗^: *p* = 0.0001,  ^∗∗∗∗^: *p* < 0.0001.

In septic mice, EPS treatment led to a significant reduction in CFU levels in both the peritoneum (Figure 6A) and peripheral blood (Figure 6B) compared to those in the CLP group. Our findings demonstrate improved control of microbial dissemination into the bloodstream following polysaccharide administration.

### 3.7. Effect of EPS Treatment on Survival in Septic Mice

**Figure 7:**
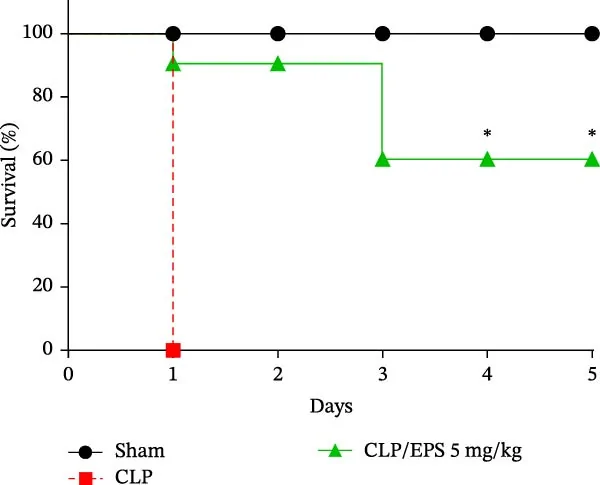
EPS increased survival rate. EPS treatment increased survival to 80% by the second day of observation and 60% by the fifth day of observation. Animals were divided into three groups—Sham: Sham‐operated mice without sepsis. CLP: Untreated mice with sepsis induced by the CLP model. CLP/EPS: Mice treated with EPS (5 mg/kg) orally for 15 days before sepsis induction. Results are expressed as the mean ± standard deviation (SD) (*n* = 5). The normality of the data was assessed using the D’Agostino–Pearson test. Statistical analysis was performed using ANOVA with Tukey’s post‐hoc test in GraphPad Prism Version 8.0.2. Differences were considered statistically significant when *p* < 0.05. For survival analysis, Kaplan–Meier curves were used, and the log‐rank test was employed to compare them. Asterisks ( ^∗^) denote a significant difference compared to the CLP group.  ^∗^: *p* < 0.0001.

All mice in the control CLP group (100%) died within 1 day following the induction of lethal sepsis. In contrast, all Sham group mice (100%) survived. Notably, 60% of mice in the CLP/EPS group survived until the 5th day after sepsis induction (Figure 7).

### 3.8. Protein–Protein Interaction Network Analysis

**Figure 8:**
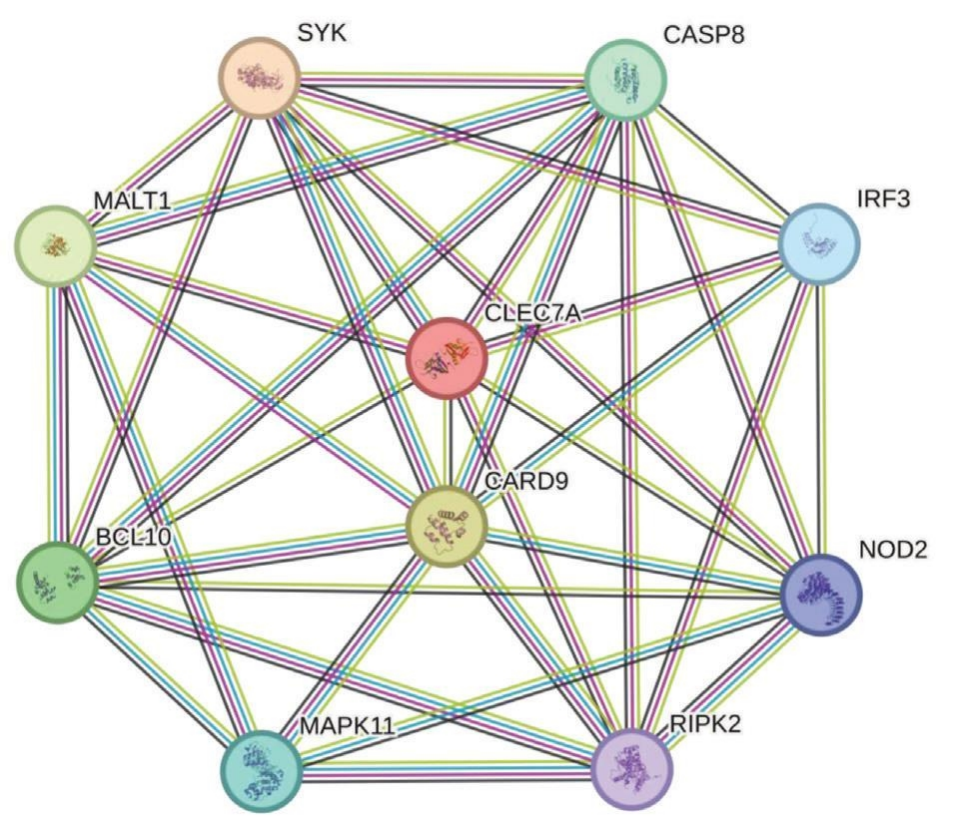
Predicted protein interaction network for β‐glucan receptor, Dectin‐1, signaling). Proteins potentially involved in the immune response to β‐glucan were analyzed using the STRING database (v12, *Mus musculus*). Lines indicate known or predicted functional associations (confidence score > 0.4). The network includes key Dectin‐1 (CLEC7A) pathway components (Syk, Card9, Bcl10, Malt1), transcription factor (IRF3), p38 MAPK proteins (Mapk11), and the bacterial sensor Nod2 with its adaptor Ripk2. This model suggests possible signaling routes for EPS‐mediated effects but does not show measured changes in protein expression or activity after treatment.

In living cells, proteins do not function in isolation; instead, they establish intricate networks of functional interactions that underpin cellular processes. Considering the important role of β‐glucan described in the EPS composition and to further elucidate the interaction of Dectin‐1 with its associated proteins and their impact on cellular functions, we performed a protein–protein interaction network analysis using the STRING system (https://string-db.org/). The results were used to generate an interactome (Figure 8).

STRING analysis revealed a significant protein association network. The central cluster identified included proteins involved in the immune response, specifically in cytokine production (Syk, Card9, Malt1, Bcl10, Casp8, Mapk11, Mapk14, Irf3, Nod2, and Ripk2). Furthermore, the analysis also highlighted other biological processes in which these proteins participate, namely, the LPS‐mediated signaling pathway, the response to muramyl dipeptide, and the stress‐activated MAPK cascade. This network analysis is predictive and hypothesis‐generating and does not demonstrate experimental activation, inhibition, or regulation of these pathways in vivo following EPS treatment.

## 4. Discussion and Conclusion

Sepsis involves a complex immune response characterized by both proinflammatory and immunosuppressive alterations. This includes a significant increase in the production of proinflammatory cytokines such as IL‐1, IL‐2, IL‐6, and TNF‐α, as well as chemokines that help mobilize immune cells to the infection site [25, 26]. During this process, which initially presents as an intense inflammatory response, a subsequent phase of immunosuppression can develop. Here, the function of immune cells becomes compromised, predisposing the individual to a numerical loss and/or functional impairment of T cells. This contributes to the worsening clinical outcomes observed in septic patients [27].

Numerous studies have consistently shown that lymphocyte counts decrease in the initial phase of sepsis, a phenomenon associated with increased mortality in septic patients. Drewry et al. [28] observed that persistent severe lymphopenia (absolute lymphocyte count ≤0.6 × 10^3^ cells/μL on the fourth day post‐sepsis diagnosis) is associated with an increased risk of mortality. Similarly, Girardot et al. [29] identified lymphopenia as a predictor of mortality, demonstrating a higher risk of death in septic shock patients admitted to the ICU. This finding is further corroborated by Vahedi et al. [30], who also highlighted lymphocytopenia as a predictor of mortality in septic patients. In another study, Jie Jiang et al. [31] demonstrated that lymphocytopenia is prevalent in most severe bacterial infections, even when neutrophil counts are not necessarily elevated. In such cases, patients with neutropenia are even more vulnerable to invasive infections that can rapidly progress to septic shock and death [32]. Our results, presented in Figure 2A, suggest that EPS treatment (CLP/EPS) significantly increased the number of circulating lymphocytes, suggesting a potential protective effect. This increase may prevent the lymphopenia frequently associated with high mortality in sepsis. A similar result was observed for granulocyte counts. This rise is likely related to the critical functions that granulocytes, particularly neutrophils, perform during sepsis, including rapid mobilization from the bloodstream to infection sites [33, 34]. The increased absolute number of granulocytes observed in the present study suggests a possible increase in neutrophil proliferation, which is a key cell in the innate immune response during infectious and inflammatory processes. While activated neutrophils can release neutrophil extracellular traps (NETs), structures crucial for pathogen containment, their unregulated production can contribute to tissue and vascular damage [35, 36]. Excessive NET formation can be linked to the development of vasculitis, an inflammatory condition of blood vessels that can lead to vascular compromise [37]. However, in our current study, we observed the absence of histopathological signs of severe inflammation and the preservation of pulmonary architecture, suggesting potential control of neutrophilic activation. One factor that might have contributed to this outcome is TNF‐α inhibition. Although a beneficial cytokine, dysregulated TNF‐α can lead to pathological conditions. In essence, TNF‐α‐mediated modulation of neutrophilic activity may have prevented the unregulated formation of NETs and, consequently, the development of vasculitis, thereby protecting tissues from collateral damage. This finding aligns with studies highlighting the role of cytokines in regulating NET release and maintaining tissue homeostasis [38, 39]. Alves‐Filho et al. [40] highlighted the critical role of neutrophils in sepsis resolution, demonstrating that disease severity is associated with dysregulated innate immune responses in a TLR‐4‐dependent manner as this phenomenon was absent in TLR‐4 knockout mice. In this context, EPS treatment in the present study was associated with improved outcomes, suggesting a modulatory effect on the host immune response and decreased susceptibility to infection.

Beyond lymphocytes and granulocytes, platelets are another crucial component of the immune response and are significantly affected during sepsis. Thrombocytopenia, or a decrease in the platelet count, occurs in septic individuals due to multiple factors, including blood dilution, decreased production, excessive consumption, and/or destruction of platelets [41, 42]. This phenomenon serves as an indicator of sepsis severity as platelets play a critical role in both the inflammatory response and coagulation during infection. Platelets are not only essential for hemostasis but also actively participate in the immune response by promoting the recruitment of neutrophils [43]. In sepsis, platelet activation occurs through direct interactions with pathogens, the inflammatory response, and coagulation cascades, all of which are triggered by endothelial tissue damage [41, 42]. However, excessive activation and consumption of platelets can lead to a significant reduction in their numbers, worsening tissue perfusion, increasing the risk of bleeding, and contributing to organ dysfunction. Consequently, thrombocytopenia becomes a negative prognostic marker in sepsis [44]. Our findings indicate that EPS treatment did not cause platelet depletion (Figure 2B), suggesting that it does not interfere with the mechanisms that ensure vascular integrity and prevent hemorrhage.

Our analysis of cellularity across various tissues revealed significant differences in immune responses between the treated (CLP/EPS) and untreated (CLP) groups. In the bone marrow (Figure 3A), reductions in cell numbers were observed in both groups, likely reflecting the mobilization of cells to the infectious focus—a common and expected phenomenon in sepsis [34]. In the spleen (Figure 3B), the increased cellularity in the CLP group could be linked to an exacerbated immune response, characteristic of the hyperinflammation seen in sepsis. Conversely, the reduction in cellularity in the CLP/EPS group suggests a potential modulatory effect of polysaccharides controlling cell migration and activation. In the peritoneum (Figure 3C), the primary site of infection in the CLP model, the increase in cells in the CLP group reflects the massive recruitment of leukocytes to the site. In contrast, the treated group showed a decrease, suggesting that EPS may have limited excessive cellular recruitment. No statistically significant difference in lymph node cellularity was observed among the groups (Figure 3D).

Sepsis is known to induce injury and malfunction in multiple organs, with the lungs being among the most severely affected. Sepsis‐induced lung injury is strongly associated with morbidity and mortality, primarily driven by the disruption of the endothelial barrier [45]. Other contributing factors to lung injury are associated with acute respiratory distress syndrome (ARDS), which is characterized by an exacerbated inflammatory response in the lungs, leading to pulmonary edema and, consequently, respiratory failure [46]. In our histological analysis of the lung (Figure 4 and Table 1), we observed no signs indicative of endothelial disruption in the EPS‐treated animals. Furthermore, the results show that animals treated with polysaccharides (CLP/EPS) exhibited characteristics similar to those of uninfected animals (Sham). This protective effect becomes even more evident when compared to septic animals (CLP), which displayed moderate to severe congestion and potential alveolar infiltration. These findings strongly suggest that EPS treatment preserves normal pulmonary architecture, thereby preventing endothelial barrier rupture and pulmonary edema formation, both of which are significant factors associated with mortality in sepsis [45].

Cytokines play a crucial role in the pathogenesis of sepsis, acting as key mediators of the host’s inflammatory response to infection. In sepsis, there is an excessive release of proinflammatory mediators, which contribute to tissue damage [47]. However, a phase of immunosuppression also occurs, characterized in part by the presence of anti‐inflammatory mediators [48]. Our study found that treatment with EPS from *A. auricula* maintained control over important inflammatory cytokines, specifically reducing TNF‐α, IFN‐γ, and MCP‐1. It also suppressed the production of the anti‐inflammatory cytokine, IL‐10. Furthermore, this treatment stimulated IL‐6 production without affecting IL‐12 levels (Figure 5).

TNF‐α is a pivotal cytokine that initiates cascades of other inflammatory cytokine production, playing a crucial role in the onset of the immune response [49]. However, its role can be either detrimental or protective, depending on the experimental model. Early studies correlated increased TNF‐α secretion with elevated mortality following LPS injection or *E. coli* infection [49, 50]. Other research has yielded similar results [51–53]. Our current study demonstrated that EPS treatment reduced TNF‐α production (Figure 5A). This effect likely contributed to improved animal survival and prevented tissue damage that can arise from excessive TNF‐α levels.

IFN‐γ is a cytokine regulated by the action of IL‐12 and IL‐18, which promote the activation of macrophage effector functions, leading to pathogen elimination via the production of nitric oxide (NO) and reactive oxygen species (ROS) [54, 55]. In sepsis, high levels of IFN‐γ, as well as its administration after CLP, are associated with the intensification of inflammation and, consequently, increased mortality [56]. Conversely, the blockade or reduction of IFN‐γ is associated with attenuated inflammation and increased survival [57–59]. In animals treated with EPS, IFN‐γ production was reduced (Figure 5B).

MCP‐1 is a chemokine frequently associated with the worsening of sepsis and the development of multiple organ dysfunctions [60]. This deterioration occurs due to the excessive recruitment of immune cells to infection sites and the release of tissue‐damaging mediators, such as pulmonary myeloperoxidase (MPO). Ramnath et al. [61] observed a direct relationship between decreased MCP‐1 and MPO levels and improvements in histological and hepatic analyses in CLP and LPS models after treatment with MCP‐1 blockers. The results, consistent with the aforementioned study, indicate a reduction in serum MCP‐1 levels, which correlates with the histological analysis of the lung (Figure 5C). This suggests a possible protective effect against pulmonary damage, thereby corroborating the findings of Ramnath et al. [61].

IL‐6 is a cytokine that can exert either beneficial or detrimental effects on survival during bacterial infections depending on the study model. It has been observed to be beneficial in some contexts [62, 63] while being harmful in others [64]. For instance, Dalrymple et al. [62] found that IL‐6‐deficient mice exhibited increased susceptibility to *E. coli* infection, with higher mortality and elevated tissue CFUs. Similarly, van der Poll et al. [63] reported that IL‐6 knockout (IL‐6 KO^−/−^) mice exhibited impaired defenses against *S. pneumoniae*‐induced infection, leading to increased mortality and higher lung CFUs. McGhee et al. [65] observed an increase in IL‐6 production by lymphocytes from mice pretreated with β‐glucan after LPS stimulation [65]. Their study examined the role of IL‐6 in modulating apoptosis in these cells. This concept is further supported by Naseem et al. [66], who reported a reduction in the percentage of apoptotic peripheral blood mononuclear cells (PBMCs) following IL‐6 stimulation. Our findings, consistent with earlier studies, indicate an increase in serum IL‐6 levels in EPS‐treated animals (Figure 5D). This increase is likely associated with the prevention of lymphopenia in sepsis, thereby contributing to improved outcomes.

β‐glucans are frequently highlighted in the literature for their low toxicity [67–69] and their antimicrobial properties [70], which enable their use in pathogen eradication. Our results demonstrated that EPS reduced CFUs at the infectious focus within the peritoneal cavity (Figure 6A) and improved control of microbial dissemination into the bloodstream (Figure 6B), thereby preventing dissemination to distant tissues. Harriett et al. [71] conducted a similar study, observing that immunization with purified β‐glucan preparations from *S. cerevisiae* (1 dose of 1–4 mg, administered intraperitoneally 7–14 days before sepsis) induced protection against *C. albicans*, *S. aureus*, and *E. coli*. This treatment resulted in an 80% survival rate up to the 10th day after sepsis induction. In our current work, we demonstrated an increased survival rate in animals treated with EPS, with an 80% survival rate by the second day of observation and 60% from the third to the fifth day of observation (Figure 7). This corroborates the data from Harriett et al. [71] and confirms that EPS treatment modulated immunological parameters, as demonstrated, inducing protection against polymicrobial sepsis and providing increased survival.

The EPS from *A. auricula* is composed of α‐ and β‐glucan and fucogalactomannans. The modulation of intestinal inflammation and the microbiota was dependent on Dectin‐1 expression, indicating that glucans are the primary polysaccharides in modulating the host immune response during experimental colitis [17]. To explore the signaling pathways involved in the protection offered by *A. auricula* EPS, which is rich in β‐glucan, we conducted a predictive protein–protein interaction network analysis using the STRING database. This in silico modeling identifies established functional connections but does not measure changes in protein expression or activity. The generated interactome pointed (Figure 8) out a central cluster of proteins essential for immune signaling. This includes Dectin‐1 (CLEC7A), Syk, CARD9, Bcl10, Malt1, IRF3 transcription factor, p38 MAPK (Mapk11), and the bacterial sensor Nod2 with its adaptor Ripk2. This network suggests that the β‐glucan (part of EPS) may signal through the Dectin‐1‐Syk‐CARD9 axis, impacting downstream proinflammatory transcription factors and MAPK pathways. The involvement of Nod2/Ripk2 indicates a possible interaction that could prepare leukocytes for improved antibacterial responses. This offers a possible explanation for the observed reduction in the bacterial load in the peritoneum and blood and changes in cytokine production. Future research is needed to measure the phosphorylation state and expression levels of these key proteins to confirm their specific activation or inhibition after EPS treatment.

The results presented in this study demonstrate that the impact of polysaccharide administration, likely EPS from *A. auricula*, in a septic model, is remarkably diverse. Treatment with EPS from *A. auricula* increased survival through several mechanisms. These include an elevated absolute number of leukocytes and granulocytes in peripheral blood, indicating a more robust immune response against the infectious agent. We also observed a significant reduction in CFUs in both peripheral blood and the infectious focus, highlighting improved control over bacterial dissemination. Furthermore, preservation of pulmonary architecture was evident, reflecting reduced inflammatory tissue damage and prevention of acute lung injury.

Even with our present study, there are limitations for translational research purposes. However, translational potential is increasingly supported through growing preclinical and clinical evidence regarding β‐glucans. Clinically, β‐glucans from fungi are widely used, safe oral nutritional supplements with established immunomodulatory effects, including a reduction in the incidence of upper respiratory infections. Established safety in healthy and clinical populations, combined with active commercial production, significantly decreases the risks of initial translational steps. Future studies with *A. auricula* EPS will be important for detailed pharmacokinetics, formulation optimization, and efficacy trials in general and at‐risk populations, building directly on this foundation of prior β‐glucan research to accelerate its development as a potential therapeutic adjunct in sepsis.

Given these findings, our data suggest that administering EPS from *A. auricula* can mitigate key factors that exacerbate sepsis and increase mortality risk. Furthermore, preadministration of β‐glucans, prior to the development of sepsis, may be particularly beneficial. This is because it primes and enhances the immune system, enabling it to respond more efficiently to polymicrobial infections. Thus, the possibility of using β‐glucans as therapeutic agents and/or adjuvants in the management of sepsis represents an innovative approach, aligning prevention with alternative therapy to improve clinical outcomes for septic patients.

## Funding

This study was supported by the Conselho Nacional de Desenvolvimento Científico e Tecnológico (Grant 305584/2023–5), the Fundação de Apoio à Pesquisa do Distrito Federal (Grants 193.00000029/2019‐53 and 00193.00002346/2022‐18, the Coordenação de Aperfeiçoamento de Pessoal de Nível Superior (Grant 405934/2022‐0), The National Institute of Science and Technology—INCT Funvir, and the DPI/BCE University of Brasília, Brazil (Edital Number 001/2025 DPI/BCE/UnB). This study was financed in part by the Coordenação de Aperfeiçoamento de Pessoal de Nível Superior – Brasil (CAPES) – Finance Code 001. CNPq (Pró‐Amazônia/CNPq process 445615/2024‐9).

## Conflicts of Interest

The authors declare no conflicts of interest.

## Data Availability

The article data supporting this study are from previously reported studies and datasets, which have been cited. The processed data are available from the corresponding author upon request.

## References

[1] Singer M. , Deutschman C. S. , and Seymour C. W. , et al.The Third International Consensus Definitions for Sepsis and Septic Shock (Sepsis-3), Journal of the American Medical Association. (2016) 315, no. 8, 801–810, 10.1001/jama.2016.0287. 26903338 PMC4968574

[2] Levy M. M. , Fink M. P. , and Marshall J. C. , et al.SCCM/ESICM/ACCP/ATS/SIS International Sepsis Definitions Conference, Critical Care Medicine. (2003) 31, no. 4, 1250–1256, 10.1097/01.CCM.0000050454.01978.3B. 12682500

[3] Rudd K. E. , Johnson S. C. , and Agesa K. M. , et al.Global, Regional, and National Sepsis Incidence and Mortality, 1990-2017: Analysis for the Global Burden of Disease Study, The Lancet. (2020) 395, no. 10219, 200–211, 10.1016/S0140-6736(19)32989-7. PMC6970225 31954465

[4] Evans L. , Rhodes A. , and Alhazzani W. , et al.Surviving Sepsis Campaign: International Guidelines for Management of Sepsis and Septic Shock 2021, Intensive Care Medicine. (2021) 47, no. 11, 1181–1247, 10.1007/s00134-021-06506-y. 34599691 PMC8486643

[5] Parrillo J. and dellinger R. , Critical Care Medicine: Principles of Diagnosis and Management in the Adult, 2007, Mosby.

[6] Vincent J. L. , Sakr Y. , and Singer M. , et al.Prevalence and Outcomes of Infection Among Patients in Intensive Care Units in 2017, Journal of the American Medical Association. (2020) 323, no. 15, 1478–1487, 10.1001/jama.2020.2717. 32207816 PMC7093816

[7] Paoli C. J. , Reynolds M. A. , Sinha M. , Gitlin M. , and Crouser E. , Epidemiology and Costs of Sepsis in the United States-An Analysis Based on Timing of Diagnosis and Severity Level, Critical Care Medicine. (2018) 46, no. 12, 1889–1897, 10.1097/CCM.0000000000003342. 30048332 PMC6250243

[8] Fleischmann C. , Scherag A. , and Adhikari N. K. , et al.Assessment of Global Incidence and Mortality of Hospital-Treated Sepsis, American Journal of Respiratory and Critical Care Medicine. (2016) 193, no. 3, 259–272, 10.1164/rccm.201504-0781OC. 26414292

[9] Iwashyna T. J. , Ely E. W. , Smith D. M. , and Langa K. M. , Long-term Cognitive Impairment and Functional Disability Among Survivors of Severe Sepsis, Journal of the American Medical Association. (2010) 304, no. 16, 1787–1794, 10.1001/jama.2010.1553. 20978258 PMC3345288

[10] La Via L. , Sangiorgio G. , and Stefani S. , et al.The Global Burden of Sepsis and Septic Shock, Epidemiologia. (2024) 5, no. 3, 456–478, 10.3390/epidemiologia5030032. 39189251 PMC11348270

[11] Vetvicka V. , Vannucci L. , and Sima P. , β-Glucan as a New Tool in Vaccine Development, Scandinavian Journal of Immunology. (2020) 91, no. 2, 10.1111/sji.12833, e12833. 31544248

[12] Ciecierska A. , Drywień M. E. , Hamulka J. , and Sadkowski T. , Nutraceutical Functions of Beta-Glucans in Human Nutrition, Roczniki Państwowego Zakładu Higieny. (2019) 70, no. 4, 315–324, 10.32394/rpzh.2019.0082. 31960663

[13] Vlassopoulou M. , Yannakoulia M. , Pletsa V. , Zervakis G. I. , and Kyriacou A. , Effects of Fungal Beta-Glucans on Health - A Systematic Review of Randomized Controlled Trials, Food and Function. (2021) 12, no. 8, 3366–3380, 10.1039/D1FO00122A. 33876798

[14] Walachowski S. , Breyne K. , and Secher T. , et al.Oral Supplementation With Yeast β-Glucans Improves the Resolution of *Escherichia coli*-Associated Inflammatory Responses Independently of Monocyte/Macrophage Immune Training, Frontiers in Immunology. (2022) 13, 10.3389/fimmu.2022.1086413, 1086413. 36605196 PMC9809295

[15] Saeed S. , Quintin J. , and Kerstens H. H. , et al.Epigenetic Programming of Monocyte-to-Macrophage Differentiation and Trained Innate Immunity, Science. (2014) 345, no. 6204, 10.1126/science.1251086, 1251086. 25258085 PMC4242194

[16] Quintin J. , Saeed S. , and Martens J. H. A. , et al. *Candida albicans* Infection Affords Protection Against Reinfection via Functional Reprogramming of Monocytes, Cell Host and Microbe. (2012) 12, no. 2, 223–232, 10.1016/j.chom.2012.06.006. 22901542 PMC3864037

[17] Coelho L. C. , Favilla L. D. , de Castro T. B. , Leal M. C. B. D. M. , Hoffmann C. , and Bocca A. L. , Auricularia Auricula’s Exopolysaccharide Mitigates DSS-Induced Colitis Through Dectin–1-Mediated Immunomodulation and Microbiota Remodeling, Pharmaceuticals. (2025) 18, no. 8, 10.3390/ph18081085, 1085. 40872478 PMC12389670

[18] Basso A. M. M. , De Castro R. J. A. , and de Castro T. B. , et al.Immunomodulatory Activity of β-Glucan-Containing Exopolysaccharides From Auricularia Auricular in Phagocytes and Mice Infected With *Cryptococcus neoformans* , Medical Mycology. (2020) 58, no. 2, 227–239, 10.1093/mmy/myz042. 31095342

[19] Benjamim C. F. , Ferreira S. H. , and Cunha F. D. Q. , Role of Nitric Oxide in the Failure of Neutrophil Migration in Sepsis, The Journal of Infectious Diseases. (2000) 182, no. 1, 214–223, 10.1086/315682. 10882600

[20] Oliveira A. S. , Nascimento J. R. , and Trovão L. O. , et al.The Anti-Inflammatory Activity of *Anacardium occidentale* L. Increases the Lifespan of Diabetic Mice With Lethal Sepsis, Journal of Ethnopharmacology. (2019) 236, 345–353, 10.1016/j.jep.2019.03.014. 30872173

[21] Sousa N. , Figueiredo I. , and Araújo M. , et al. *Punica granatum* Leaf Lipophilic Fraction Favours Mortality by Attenuating Inflammation in Mice With Polymicrobial Sepsis, Journal of Pharmacy and Pharmacology. (2019) 7, no. 7, 355–367, 10.17265/2328-2150/2019.07.002.

[22] de O Trovão L. , Rodrigues L. D. S. , and Mendes P. M. , et al.The Immunomodulatory Activity of *Punica granatum* L. Peel Extract Increases the Lifespan of Mice with Lethal Sepsis, Journal of Immunology Research. (2023) 2023, 10.1155/2023/2868707, 2868707. 37621924 PMC10447006

[23] Hodge G. , Hodge S. , and Haslam R. , et al.Rapid Simultaneous Measurement of Multiple Cytokines Using 100 Microl Sample Volumes-Association With Neonatal Sepsis, Clinical and Experimental Immunology. (2004) 137, no. 2, 402–407, 10.1111/j.1365-2249.2004.02529.x. 15270859 PMC1809114

[24] Szklarczyk D. , Gable A. L. , and Lyon D. , et al.STRING v11: Protein-Protein Association Networks With Increased Coverage, Supporting Functional Discovery in Genome-Wide Experimental Datasets, Nucleic Acids Research. (2019) 47, no. D1, D607–D613, 10.1093/nar/gky1131. 30476243 PMC6323986

[25] Hotchkiss R. S. , Monneret G. , and Payen D. , Sepsis-Induced Immunosuppression: From Cellular Dysfunctions to Immunotherapy, Nature Reviews Immunology. (2013) 13, no. 12, 862–874, 10.1038/nri3552. PMC4077177 24232462

[26] van der Poll T. , van de Veerdonk F. L. , Scicluna B. P. , and Netea M. G. , The Immunopathology of Sepsis and Potential Therapeutic Targets, Nature Reviews Immunology. (2017) 17, no. 7, 407–420, 10.1038/nri.2017.36. 28436424

[27] Venet F. and Monneret G. , Advances in the Understanding and Treatment of Sepsis-Induced Immunosuppression, Nature Reviews Nephrology. (2018) 14, no. 2, 121–137, 10.1038/nrneph.2017.165. 29225343

[28] Drewry A. M. , Samra N. , Skrupky L. P. , Fuller B. M. , Compton S. M. , and Hotchkiss R. S. , Persistent Lymphopenia After Diagnosis of Sepsis Predicts Mortality, Shock. (2014) 42, no. 5, 383–391, 10.1097/SHK.0000000000000234. 25051284 PMC4362626

[29] Girardot T. , Rimmelé T. , Venet F. , and Monneret G. , Apoptosis-Induced Lymphopenia in Sepsis and Other Severe Injuries, Apoptosis. (2017) 22, no. 2, 295–305, 10.1007/s10495-016-1325-3. 27812767

[30] Sheikh Motahar Vahedi H. , Bagheri A. , Jahanshir A. , Seyedhosseini J. , and Vahidi E. , Association of Lymphopenia With Short Term Outcomes of Sepsis Patients; a Brief Report, Archives of Academic Emergency Medicine. (2019) 7, no. 1, e14. 30847449 PMC6377227

[31] Jiang J. , Du H. , and Su Y. , et al.Nonviral Infection-Related Lymphocytopenia for the Prediction of Adult Sepsis and Its Persistence Indicates a Higher Mortality, Medicine. (2019) 98, no. 29, 10.1097/MD.0000000000016535, e16535. 31335735 PMC6708870

[32] Guillotin F. , Aubert L. , and Benguerfi S. , et al.Neutropenic Sepsis and Septic Shock in ICU Patients: A Single-Center Experience over the Last Decade, PLoS One. (2025) 20, no. 10, 10.1371/journal.pone.0334511. PMC12527136 41091692

[33] Delano M. J. and Ward P. A. , Sepsis-Induced Immune Dysfunction: Can Immune Therapies Reduce Mortality?, Journal of Clinical Investigation. (2016) 126, no. 1, 23–31, 10.1172/JCI82224. 26727230 PMC4701539

[34] Delano M. J. and Ward P. A. , The Immune System’s Role in Sepsis Progression, Resolution, and Long-Term Outcome, Immunological Reviews. (2016) 274, no. 1, 330–353, 10.1111/imr.12499. 27782333 PMC5111634

[35] Brinkmann V. , Reichard U. , and Goosmann C. , et al.Neutrophil Extracellular Traps Kill Bacteria, Science. (2004) 303, no. 5663, 1532–1535, 10.1126/science.1092385. 15001782

[36] Papayannopoulos V. , Neutrophil Extracellular Traps in Immunity and Disease, Nature Reviews Immunology. (2018) 18, no. 2, 134–147, 10.1038/nri.2017.105. 28990587

[37] Kessenbrock K. , Krumbholz M. , and Schönermarck U. , et al.Netting Neutrophils in Autoimmune Small-Vessel Vasculitis, Nature Medicine. (2009) 15, no. 6, 623–625, 10.1038/nm.1959. PMC2760083 19448636

[38] Kaplan M. J. and Radic M. , Neutrophil Extracellular Traps: Double-Edged Swords of Innate Immunity, The Journal of Immunology. (2012) 189, no. 6, 2689–2695, 10.4049/jimmunol.1201719. 22956760 PMC3439169

[39] Masuda S. , Nakazawa D. , and Shida H. , et al.NETosis Markers: Quest for Specific, Objective, and Quantitative Markers, Clinica Chimica Acta. (2016) 459, 89–93, 10.1016/j.cca.2016.05.029. 27259468

[40] Alves-Filho J. C. , Benjamim C. , Tavares-Murta B. M. , and Cunha F. Q. , Failure of Neutrophil Migration Toward Infectious Focus in Severe Sepsis: A Critical Event for the Outcome of This Syndrome, Memórias do Instituto Oswaldo Cruz. (2005) 100, no. suppl 1, 223–226, 10.1590/s0074-02762005000900038. 15962127

[41] Vardon-Bounes F. , Gratacap M. P. , and Groyer S. , et al.Kinetics of Mean Platelet Volume Predicts Mortality in Patients With Septic Shock, PLoS One. (2019) 14, no. 10, 10.1371/journal.pone.0223553, e0223553. 31622365 PMC6797099

[42] Vardon-Bounes F. , Ruiz S. , Gratacap M. P. , Garcia C. , Payrastre B. , and Minville V. , Platelets Are Critical Key Players in Sepsis, International Journal of Molecular Sciences. (2019) 20, no. 14, 10.3390/ijms20143494, 3494. 31315248 PMC6679237

[43] Grommes J. , Alard J. E. , and Drechsler M. , et al.Disruption of Platelet-Derived Chemokine Heteromers Prevents Neutrophil Extravasation in Acute Lung Injury, American Journal of Respiratory and Critical Care Medicine. (2012) 185, no. 6, 628–636, 10.1164/rccm.201108-1533OC. 22246174 PMC3326286

[44] Levi M. and van der Poll T. , Coagulation and Sepsis, Thrombosis Research. (2017) 149, 38–44, 10.1016/j.thromres.2016.11.007. 27886531

[45] Dolmatova E. V. , Forrester S. J. , and Wang K. , et al.Endothelial Poldip2 Regulates Sepsis-Induced Lung Injury via Rho Pathway Activation, Cardiovascular Research. (2022) 118, no. 11, 2506–2518, 10.1093/cvr/cvab295. 34528082 PMC9612795

[46] Sheu C. C. , Gong M. N. , and Zhai R. , et al.Clinical Characteristics and Outcomes of Sepsis-Related vs Non-Sepsis-Related ARDS, Chest. (2010) 138, no. 3, 559–567, 10.1378/chest.09-2933. 20507948 PMC2940067

[47] Dinarello C. A. , Proinflammatory Cytokines, Chest. (2000) 118, no. 2, 503–508, 10.1378/chest.118.2.503. 10936147

[48] Hotchkiss R. S. and Nicholson D. W. , Apoptosis and Caspases Regulate Death and Inflammation in Sepsis, Nature Reviews Immunology. (2006) 6, no. 11, 813–822, 10.1038/nri1943. 17039247

[49] Tracey K. J. , Lowry S. F. , and Fahey T. J. , et al.Cachectin/Tumor Necrosis Factor Induces Lethal Shock and Stress Hormone Responses in the Dog, Surgery Gynecology and Obstetrics. (1987) 164, no. 5, 415–422. 3576418

[50] Beutler B. , Milsark I. W. , and Cerami A. C. , Passive Immunization Against Cachectin/Tumor Necrosis Factor Protects Mice from Lethal Effect of Endotoxin, Science. (1985) 229, no. 4716, 869–871, 10.1126/science.3895437. 3895437

[51] Suffredini A. F. , Fromm R. E. , and Parker M. M. , et al.The Cardiovascular Response of Normal Humans to the Administration of Endotoxin, New England Journal of Medicine. (1989) 321, no. 5, 280–287, 10.1056/NEJM198908033210503. 2664516

[52] Taveira da Silva A. M. , Kaulbach H. C. , Chuidian F. S. , Lambert D. R. , Suffredini A. F. , and Danner R. L. , Brief Report: Shock and Multiple-Organ Dysfunction after Self-Administration of Salmonella Endotoxin, New England Journal of Medicine. (1993) 328, no. 20, 1457–1460, 10.1056/NEJM199305203282005. 8479465

[53] Waage A. , Halstensen A. , and Espevik T. , Association between Tumour Necrosis Factor in Serum and Fatal Outcome in Patients With Meningococcal Disease, The Lancet. (1987) 329, no. 8529, 355–357, 10.1016/S0140-6736(87)91728-4. 2880163

[54] Lionakis M. S. , Drummond R. A. , and Hohl T. M. , Immune Responses to Human Fungal Pathogens and Therapeutic Prospects, Nature Reviews Immunology. (2023) 23, no. 7, 433–452, 10.1038/s41577-022-00826-w. PMC9812358 36600071

[55] Romero C. R. , Herzig D. S. , and Etogo A. , et al.The Role of Interferon-γ in the Pathogenesis of Acute Intra-Abdominal Sepsis, Journal of Leukocyte Biology. (2010) 88, no. 4, 725–735, 10.1189/jlb.0509307. 20628064 PMC2974432

[56] Miles R. H. , Paxton T. P. , Dries D. J. , and Gamelli R. L. , Interferon-Gamma Increases Mortality Following Cecal Ligation and Puncture, The Journal of Trauma: Injury, Infection, and Critical Care. (1994) 36, no. 5, 607–611, 10.1097/00005373-199405000-00001. 8189458

[57] Clements J. D. , Dinges M. M. , and Schlievert P. M. , Role of T Cells and Gamma Interferon During Induction of Hypersensitivity to Lipopolysaccharide by Toxic Shock Syndrome Toxin 1 in Mice, Infection and Immunity. (2001) 69, no. 3, 1256–1264, 10.1128/IAI.69.3.1256-1264.2001. 11179286 PMC98015

[58] Yin K. , Gribbin E. , and Wang H. , Interferon-Gamma Inhibition Attenuates Lethality After Cecal Ligation and Puncture in Rats: Implication of High Mobility Group Box-1, Shock. (2005) 24, no. 4, 396–401, 10.1097/01.shk.0000175556.03300.c6. 16205327

[59] Yin K. , Hock C. E. , Lai P. S. , Ross J. T. , and Yue G. , Role of Interferon-Gamma in Lung Inflammation following Cecal Ligation and Puncture in Rats, Shock. (1999) 12, no. 3, 215–221, 10.1097/00024382-199909000-00008. 10485600

[60] Wang T. , Dai H. , Wan N. , Moore Y. , and Dai Z. , The Role for Monocyte Chemoattractant Protein-1 in the Generation and Function of Memory CD8+ T Cells, The Journal of Immunology. (2008) 180, no. 5, 2886–2893, 10.4049/jimmunol.180.5.2886. 18292510

[61] Ramnath R. D. , Ng S. W. , Guglielmotti A. , and Bhatia M. , Role of MCP-1 in Endotoxemia and Sepsis, International Immunopharmacology. (2008) 8, no. 6, 810–818, 10.1016/j.intimp.2008.01.033. 18442784

[62] Dalrymple S. A. , Slattery R. , Aud D. M. , Krishna M. , Lucian L. A. , and Murray R. , Interleukin-6 is Required for a Protective Immune Response to Systemic *Escherichia coli* Infection, Infection and Immunity. (1996) 64, no. 8, 3231–3235, 10.1128/iai.64.8.3231-3235.1996. 8757858 PMC174212

[63] van der Poll T. , Keogh C. V. , Guirao X. , Buurman W. A. , Kopf M. , and Lowry S. F. , Interleukin-6 Gene-Deficient Mice Show Impaired Defense Against Pneumococcal Pneumonia, The Journal of Infectious Diseases. (1997) 176, no. 2, 439–444, 10.1086/514062. 9237710

[64] Riedemann N. C. , Neff T. A. , and Guo R. F. , et al.Protective Effects of IL-6 Blockade in Sepsis Are Linked to Reduced C5a Receptor Expression, The Journal of Immunology. (2003) 170, no. 1, 503–507, 10.4049/jimmunol.170.1.503. 12496437

[65] McGhee J. R. , Soltys J. , and Quinn M. T. , Modulation of Endotoxin- and Enterotoxin-Induced Cytokine Release by In Vivo Treatment With Beta-(1,6)-Branched Beta-(1,3)-Glucan, Infection and Immunity. (1999) 67, no. 1, 244–252, 10.1128/IAI.67.1.244-252.1999. 9864222 PMC96303

[66] Naseem S. , Manzoor S. , Javed A. , and Abbas S. , Interleukin-6 Rescues Lymphocyte From Apoptosis and Exhaustion Induced by Chronic Hepatitis C Virus Infection, Viral Immunology. (2018) 31, no. 9, 624–631, 10.1089/vim.2018.0045. 30222516

[67] Cardenas F. I. , Mauguen A. , and Cheung I. Y. , et al.Phase I Trial of Oral Yeast-Derived β-Glucan to Enhance Anti-GD2 Immunotherapy of Resistant High-Risk Neuroblastoma, Cancers. (2021) 13, no. 24, 10.3390/cancers13246265, 6265. 34944886 PMC8699451

[68] Preece K. E. , Glávits R. , and Murbach T. S. , et al.Assessment of Toxicological Potential of Sodium Carboxymethyl Beta-Glucan, a Novel Beta-Glucan, Food and Chemical Toxicology. (2021) 152, 10.1016/j.fct.2021.112226, 112226. 33905759

[69] Tatli Seven P. , Iflazoglu Mutlu S. , Seven I. , Arkali G. , Ozer Kaya S. , and Kanmaz O. E. , Protective Role of Yeast Beta-Glucan on Lead Acetate-Induced Hepatic and Reproductive Toxicity in Rats, Environmental Science and Pollution Research. (2021) 28, no. 38, 53668–53678, 10.1007/s11356-021-14398-0. 34036504

[70] Wzorek-Łyczko K. , Woźniak W. , Piwowarczyk A. , and Kuchar E. , The Anti-Infective Effect of β-Glucans in Children, International Journal for Vitamin and Nutrition Research. (2024) 94, no. 3-4, 296–307, 10.1024/0300-9831/a000793. 37779363

[71] Harriett A. J. , Esher Righi S. , Lilly E. A. , Fidel P.Jr., and Noverr M. C. , Efficacy of *Candida dubliniensis* and Fungal β-Glucans in Inducing Trained Innate Immune Protection Against Inducers of Sepsis, Frontiers in Cellular and Infection Microbiology. (2022) 12, 10.3389/fcimb.2022.898030, 898030. 35770067 PMC9234138

